# The Suicidal Intrusions Attributes Scale (SINAS): a new tool measuring suicidal intrusions

**DOI:** 10.3389/fpsyt.2023.1158340

**Published:** 2023-07-05

**Authors:** Jaël S. van Bentum, Ad J. F. M. Kerkhof, Marcus J. H. Huibers, Emily A. Holmes, Stephan de Geus, Marit Sijbrandij

**Affiliations:** ^1^Clinical, Neuro-, and Developmental Psychology, Amsterdam Public Health Research Institute, Vrije Universiteit Amsterdam, Amsterdam, Netherlands; ^2^Department of Clinical Psychology, Universiteit Utrecht, Utrecht, Netherlands; ^3^NPI Center for Personality Disorders, Arkin, Amsterdam, Netherlands; ^4^Department of Psychology, Uppsala University, Uppsala, Sweden; ^5^World Health Organization Collaborating Centre for Research and Dissemination of Psychological Interventions, Vrije Universiteit Amsterdam, Amsterdam, Netherlands

**Keywords:** suicidal imagery, intrusions, Suicidal Intrusions Attributes Scale, validation study, mental imagery, intrusiveness, suicide

## Abstract

**Introduction:**

Suicidal intrusions are uncontrollable, intrusive mental images (e. g., visualizing a future suicidal act). They may also be called suicidal “flash-forwards.” Despite the importance of integrating the assessment of suicidal intrusions into a clinical routine assessment, quick self-report screening instruments are lacking. This study describes the development of a new instrument—Suicidal Intrusions Attributes Scale (SINAS)—to assess the severity and characteristics of suicidal intrusions and examines its psychometric properties.

**Method:**

The sample included currently suicidal outpatients with elevated levels of depression recruited across mental health institutions in the Netherlands (*N* = 168). Instruments administered were 10-item SINAS, the Suicidal Ideation Attributes Scale (SIDAS), the Prospective Imagery Task (PIT), four-item Suicidal Cognitions Interview (SCI), and the Beck Depression Inventory (BDI-II).

**Results:**

An exploratory factor analysis identified a one-factor structure. The resulting SINAS demonstrated good internal consistency (Cronbach's α = 0.91) and convergent validity, as expected.

**Discussion:**

Overall, this study demonstrated acceptable levels of reliability and validity of the measure in a depressed clinical population with suicidal ideation. The SINAS may be a useful screening tool for suicidal intrusions in both research and clinical settings.

## 1. Introduction

Suicidal intrusions can be defined as vivid, uncontrollable mental images and thoughts of suicide or the aftermath of one's suicidal death (also referred to as suicidal “flash-forwards”) (1–3). The contents of these intrusions may vary from images of a future suicidal act (e.g., jumping off a cliff or taking an overdose) to the consequences of one's suicide (e.g., seeing one's own funeral or seeing the reactions of their loved ones to the news). Initially, these mental images can be perceived as comforting as they may offer a route to escape the distress one experiences (4), as indicated by the findings that they can feel as pleasant to the patient as they are distressing (1, 5). As the number of occurrences increases, the intrusions may be experienced as more adverse, distressing, inescapable, and intolerable for some patients. For other intrusions, the positivity/sense of relief associated with the intrusions (e.g., the death would feel calm and reasonable) may drive people to want to act on them (6). Suppression attempts are assumed to increase thought frequency (7), reinforcing the desire to escape the distress/or lure caused by suicidal intrusions. Suicidal intrusions differ from similar phenomena observed in those with obsessive-compulsive disorder (e.g., “aggressive” obsessions) and, in that, the latter appears to be ego-syntonic (8). To date, studies have shown that suicidal intrusions occur in various psychiatric disorders, including unipolar and bipolar (3, 9, 10) depression and borderline personality disorder (11).

Suicidal intrusions are an important phenomenon since their occurrence may promote suicidal behavior (12) and since mental imagery (as opposed to verbal thinking) can drive the motivation to act (13). Previous research studies have shown that mentally imagining an action (e.g., voting in an election) may increase the probability of engaging in that action (14). Moreover, studies have shown that mental imagery allows individuals to identify the initial barriers to realizing a particular event and adjusting behaviors accordingly (2, 11, 14). For example, a previous study found that individuals with suicidal intrusions had more severe suicidal ideation than individuals without suicidal intrusions (11). Furthermore, individuals with mental images of suicide reported greater preoccupation with suicide than suicide-related verbal thoughts (5). Previous research among adolescent psychiatric inpatients found that suicidal mental imagery was associated with a higher likelihood of suicide attempts (6), whereas among undergraduate college students, suicidal imagery was associated with an increased likelihood of both suicide plans and attempts (15). To date, clinicians often do not inquire about them (instead focus on more verbal forms of cognition) as they may fear untoward effects when asking patients about it or they lack sufficient treatment tools.

Despite the importance of integrating the assessment of suicidal intrusions into clinical routine assessment, quick self-report screening instruments are still lacking. Many studies of suicidal ideation use validated instruments such as the single suicide item in the Beck Depression Inventory-II (BDI-II) (16) and the Beck Scale of Suicidal Ideation (BSSI) (17), but none of these scales include items on suicidal mental imagery or intrusions. Therefore, research into suicidal ideation systematically underestimated the presence and importance of suicidal intrusive imagery.

To date, the Suicidal Cognitions Interview (SCI) (1) has been used in various studies (1, 2, 18, 19) to assess the presence, content, and characteristics of suicidal intrusions/cognitions in general and clinical populations. The semi-structured interview assesses verbal thoughts and mental images about suicide by asking for examples and ratings of the examples regarding their levels of distress, vividness, and time spent on the mental image. This interview provides a detailed description of suicidal cognitions and intrusions, but it is time-consuming, difficult to implement in clinical practice, and has, to the best of our knowledge, not been validated. Moreover, this interview was also developed based on the imagery experience of patients with social phobia, agoraphobia, and post-traumatic stress disorders and not on the imagery experience of suicide risk groups (1).

In response to the lack of self-report assessment tools, Ko and You (20) recently developed the Suicidal Imagery Questionnaire (SIQ). This scale consists of 10 items and is divided into two categories: spontaneous suicidal imagery (six items) and intrusive suicidal imagery (four items). A recent review by Baek et al. (21) reported good psychometric properties of the SIQ. While the instrument has been validated, it has only been administered in a non-clinical Korean population (20). Furthermore, the intrusive suicidal imagery subscale only focuses on the presence of the intrusions and does not address various relevant characteristics such as the vividness, severity, and controllability of the intrusions.

To address the shortcomings of the previously mentioned instruments, we have developed a new brief screening instrument to measure the severity of suicidal intrusions named the Suicidal Intrusion Attributes Scale (SINAS). The SINAS items address the occurrence of suicidal intrusions in the past week and are scored on a 10-point Likert scale. The scale has the same structure as the Suicidal Ideation Attributes Scale (SIDAS) (22), which is a brief screening and assessment tool to measure attributes of suicidal ideation with higher scores indicating an increased risk of suicidal behavior. In contrast to the SIDAS, the newly developed SINAS focuses explicitly on suicide-related mental images, how compelling they are, and their compulsiveness and intrusiveness. The SINAS measures six different attributes of suicidal intrusions. The first two attributes, frequency and controllability, are similar to the SIDAS attributes and touch upon the ruminative aspects of suicidal imagery (13, 18, 23). The third attribute, closeness to attempt, reflects the possible association between suicidal behavior and the severity of the suicidal intrusions in changing behavior. The fourth and fifth attributes focus on the characteristics (distress and vividness) of suicidal intrusions. The sixth and last attribute addresses the potential compulsiveness of suicidal intrusions. Thus, while the SIDAS explicitly focuses on suicidal cognitions and ideation (of a more verbal nature), the SINAS focuses on suicidal mental imagery.

This study aimed to develop a reliable, valid, and easy-to-administer self-report instrument to facilitate the screening and assessment of suicidal intrusions (i.e., taking the form of mental imagery flash-forwards) in research and clinical settings. This instrument may be used to detect suicidal intrusions across clinical populations and may be used in future studies to identify individuals at a high risk of suicide. To examine the psychometric properties of the Dutch SINAS, the instrument was administered in an outpatient population with depression and having suicidal ideation, and an exploratory factor analysis was performed. Since all attributes assess the severity of suicidal intrusions, we expected strong correlations among these attributes. Furthermore, we assessed the convergent and divergent validity of the SINAS against the SIDAS, SCI, BDI-II, and a scale of general mental imagery unrelated to suicide [Prospective Imagery Task (24)].

## 2. Methods

### 2.1. Initial item and instrument development

The first step in creating the new assessment tool (SINAS) was to generate a suitable set of items for the SINAS (25). Based on the SIDAS (22) framework, the authors created 10 items across six attributes/domains that may characterize suicidal intrusions: frequency, controllability, closeness to a suicide attempt, distress, vividness, and compulsiveness. The attributes were developed by evaluating similar domains of intrusiveness of verbal thoughts related to suicide and suicidal ideation. All these attributes together measure the umbrella term “intrusiveness.” The “closeness to attempt” item, or in other words, perceived proximity of a future suicide attempt, was included to evaluate whether higher scores on the SINAS would be associated with the pending risk of suicide in the eyes of the respondents. Similar to the SIDAS, we used a 10-point Likert scale (e.g., 0 = *not at all* to 10 = *completely*). Coach et al. (26) stated that the scales including six points or more can provide consistent responses across participants. The instructions of the SINAS offer a clear definition of a suicidal intrusion so that it is clear to the respondent that the questions are about the intrusive mental images of their suicide in the near future (as opposed to merely being exposed to pictures of suicide).

Initially, we conducted an exploratory study in a general population sample (*N* = 401). However, partly due to convenience sampling and the online setting of the study, the respondents were mostly young, relatively healthy participants with few to no suicidal ideation and no suicidal intrusions. Thus, too much skewness and too little spread prevented us from conducting any validation analyses. This redistribution in a healthy sample clarified and thus led us to the conclusion that we should focus solely on clinical samples.

### 2.2. Study design and participants

The current research used a cross-sectional quantitative research design. This study used secondary data from a large multicenter randomized clinical trial (27) that aimed to determine the occurrence of suicidal intrusions and to evaluate the effectiveness of an add-on intervention (Eye Movement Dual Task; EMDT) in reducing suicidal intrusions. The medical ethical review committee of the Amsterdam UMC (protocol number 2017.237) approved the study protocol.

The study was conducted at eight mental health institutions in the Netherlands and included 168 outpatients currently experiencing suicidal ideation. The inclusion criteria were as follows: (a) a score of ≥1 on the Suicidal Ideation Attributes Scale (SIDAS) (22), (b) age being 18 years or older, (c) adequate proficiency in the Dutch language, and (d) currently receiving outpatient treatment for their depressive symptoms [see Table 1 for an overview of Axis-I disorders according to the Mini International Neuropsychiatric Interview (MINI)] (28) at one of the participating mental health institutions. An exclusion criterion was having a *current* psychotic episode according to the DSM-IV criteria as confirmed by the MINI.

**Table 1:** Descriptive characteristics and severity of suicidal intrusion in clinical population sample (*N* = 168).

**Demographic characteristics**	***N***	%^*^	***M***	**SD**	**Range**
**Sex**					
Women	101	60.1			
Men	67	39.9			
Age			35.30	13.14	18.14–70.11
**Marital status**					
Single	92	54.8			
In a relationship	27	16.1			
Married	40	23.8			
Divorced	8	4.8			
Widowed	1	0.6			
**Education** ^a^					
Lower	12	7.1			
Middle	132	78.6			
Higher	24	14.3			
**DSM-IV diagnoses**					
Major depressive disorder	130	84.4			
Dysthymia	14	8.7			
Posttraumatic stress disorder	28	18.3			
Panic disorder	58	37.9			
Generalized anxiety disorder	36	23.5			
Social phobia	34	22.2			
Agoraphobia	30	19.6			
Obsessive-compulsive disorder	13	8.5			
Psychotic disorder	4	2.6			
Alcohol dependence	9	5.9			
Drugs dependence	9	5.9			
Number of comorbid Axis I disorders	3.20				
**Suicidal intrusions**					
SINAS			46.15	24.11	0–98
SCI			27.02	6.57	4–36
**Suicidal ideation**					
SIDAS			28.11	10.47	3–48
**Mental imagery**					
PIT			59.5	14.83	20–100
**Depressive symptoms**					
BDI-II			28.11	10.55	9–62

BDI-II, Beck Depression Inventory second edition; PIT, Prospective Imagery Task; SCI, Suicidal Cognitions Interview; SINAS, Suicidal Intrusions Attributes Scale; SIDAS, Suicidal Ideation Attributes Scale. Possible scores on the SIDAS ranged from 0 to 50 with higher scores indicating more severe suicidal ideation. Possible scores on the SINAS range from 0 to 100 with higher scores indicating more severe suicidal intrusions.

^a^Lower education was defined as no former education, special lower education, primary school, or practical training school. Middle-level education was defined as completing lower or higher general secondary education or intermediate vocational education. Higher education was defined as completing higher vocational education, pre-university education, or university.

^*^Valid percentages are reported.

### 2.3. Procedure

If a therapist believed that an individual was eligible to participate in the Simagery project, they were asked for their oral consent to be contacted by the research team to be informed about participating in the qualification phase. For the current analyses, we included all individuals that completed the Suicidal Cognitions Interview (SCI) (1) (*N* = 168).

Only if given permission by the individual was the individual contacted for further information and met face-to-face at a later time. Flyers were also distributed in waiting rooms, and the participant could contact their therapist if interested. Next, the research assistant met the participant, and written and oral consent was provided. Participants were screened for current suicidal ideation (SIDAS) (22), current depressive symptoms (Beck Depression Inventory-II; BDI-II) (16), and DSM-IV diagnoses with the MINI. If eligible, SCI (1) was administered. Moreover, participants were asked to complete a set of online self-report questionnaires including demographics, prospective imagery task (PIT) (29, 30), and the Suicidal Intrusions Attributes Scale (SINAS) (25).

### 2.4. Measures

#### 2.4.1. Demographics

Demographic characteristics, namely, age, sex, education, employment status, and marital status were collected.

#### 2.4.2. Suicidal intrusions

The SINAS (25) is a 10-item self-report instrument measuring the characteristics of suicidal intrusions that are mental imagery-based in nature. The SINAS is based on the methods of the Suicidal Ideation Attributes Scale (22). The items of SINAS are scored on a 10-point scale to assess the frequency, intensity, vividness, and uncontrollability of the suicidal intrusions during the previous week and the perceived nearness to a future attempt. A score of 0 corresponds to an absence of the characteristics described in the item, and a score of 10 corresponds to a strong presence of this characteristic (e.g., “how often did you experience images about your own suicide” is scored as 0 = not at all to 10 = constantly). Total scores are the sum of all items, ranging between 0 and 100. One item (item 8) is reversely scored. Higher scores indicate more severe suicidal intrusions. It takes 1 to 2 min to complete the questionnaire. The complete Dutch questionnaire, including answering options can be found in Supplementary material S1. The translated English items can be found in Table 2.

**Table 2:** Rotated factor loadings (pattern matrix) for the one-factor exploratory factor analysis of the Suicidal Intrusions Attributes Scale in the clinical population (*N* = 164).

**Question: In the past week, …**	**Attribute**	**Factor loading**
1. How often did you have mental images of your own suicide?	Frequency	0.87
2. How much control did you feel over these mental images?	Controllability	0.61
3. How close were you to a suicide attempt?	Closeness to a suicide attempt	0.65
4. To what extent have you been tormented by mental images of suicide?	Distress	0.86
5. To what extent have mental images of suicide interfered with your daily activities such as work, household, and social activities?	Distress	0.77
6. How intrusive were the mental images of suicide you experienced?	Distress	0.92
7. How vivid were the images of suicide you experienced?	Vividness	0.88
8. Could you stop these mental images if you wanted to?	Controlability	0.34
9. Have you had mental images so clear that they seemed to be real?	Vividness	0.75
10. Did you feel like you had to have such mental images, like a compulsion you couldn't escape?	Compulsiveness	0.52

Principal factor analysis using oblique rotation (Oblimin, delta = 0) was used to determine factor loadings.

The Suicidal Cognitions Interview (SCI) (1) is a 21-item structured interview that assessed the content of mental images and verbal thoughts about suicide. At the start, the terms “verbal thoughts” and “mental imagery” were explained using examples, and feedback was requested to confirm if they understood the difference. The interview assessed the content of suicidal cognitions when participants were at their most despairing moment as well as a detailed description and characteristics of their most significant suicidal mental image. For this study, the interview was back-to-back translated to Dutch, and the entire interview was administered. Four items into overall experiences with suicidal mental imagery (excluding items related to content or characteristics of the most important image) were used in the current analyses. Total scores were calculated as the sum of the items ranging from 4 to 36, with higher scores indicating a higher severity of suicidal imagery. In this study, adequate internal consistency was found for the four items (Cronbach's α = 0.69).

#### 2.4.3. Suicidal ideation

The SIDAS (22) is a self-report instrument measuring the presence and severity of suicidal ideation. It contains five items assessing the frequency, controllability, closeness to attempt, distress, and interference with daily activities on 10-point scales over the previous month (e.g., “how often have you had thoughts about suicide?” is scored as 0 = never to 10 = always). Total scores are calculated as the sum of all items and range between 0 and 50, with scores above 21 indicating a high risk of suicidal behavior. If participants scored 0 on the first item, the remaining 4 items were not administered. In the current sample, the SIDAS demonstrated acceptable internal consistency (Cronbach's α = 0.77). It takes a few minutes to complete the questionnaire.

#### 2.4.4. Prospective mental imagery

The PIT (29, 30) is a 20-item self-report instrument measuring mental imagery for future situations. The items assess the vividness of mental images about both future positive events (e.g., “you will have lots of energy and enthusiasm”) and negative events (e.g., “Someone close to you will reject you”). Participants score the vividness on a 5-point scale, from 1 (no image) to 5 (very vivid image). The total score is calculated by the sum of all the items. In this study, the scale had good internal consistency (Cronbach's α = 0.88). This tool measures the vividness of imagery of negative and positive prospective events. The reason to include this scale in the current study is to control individual differences in utilizing prospective imagery concerning non-suicidal situations.

#### 2.4.5. Clinical symptoms

The BDI-II (16) includes 21 items, consisting of four self-evaluative statements about a particular symptom of depression. The statements are scored 0 to 3, and higher scores indicate greater depression severity. Total scores range between 0 and 63 and scores above 16 indicate clinical depression. The instrument shows great reliability and validity [Dutch version (31)] and high internal consistency in a clinical population (Cronbach's α = 0.92). In the current sample, the BDI-II demonstrated good internal consistency (Cronbach's α = 0.86).

### 2.5. Data analysis

Descriptive statistics were used to explore participant characteristics (*N* = 168). Correlations between each item and the total score were evaluated using a correlation matrix (26). The items that were correlated with each other at 0.85 or greater were acknowledged as high correlations, which may indicate redundancy among the items. If this was the case, we reviewed each item and decided which of the pair to retain and which to potentially omit (26).

Cronbach's alpha was calculated for the total score to assess the instrument's internal reliability. Furthermore, a leave-one-out analysis was conducted to identify whether the reliability coefficient improved or remained acceptable with the removal of particular single items.

Next, we used an exploratory factor analysis (EFA) to investigate the factor structure of the SINAS. We checked the Kaiser–Meyer–Olkin measure of sampling adequacy (value >0.60 suggests adequate data) (32) and Bartlett's test of sphericity to analyze whether the data were suitable to conduct an EFA. To determine the number of factors, the Kaiser's rule (A unique value of 1 or higher), screen plot, parallel analyses, and interpretability were reviewed. For EFA, principal factor analysis with oblique rotation (Oblimin, delta = 0) was used.

Convergent validity was evaluated using Pearson's correlation coefficients, comparing the SINAS items to the SCI and SINAS. Convergent validity was deemed adequate for Pearson's *r* ≥ 0.3 according to Cohen's (33) guidelines. To assess divergent validity, Pearson's correlation coefficients were evaluated by comparing SINAS total scores and BDI-II and PIT. All analyses were performed using Stata (version 16.1).

## 3. Results

### 3.1. Description of the sample

Most participants were women (60.1%), and the mean age was 35.3 years (SD = 13.1). A total of 7% (*n* = 12) had no education or finished lower education (no former education or special lower education, primary or practical training school), 79% (*n* = 132) had completed middle-level education (lower or higher general secondary education or intermediate vocational education), and 14% (*n* = 24) had completed higher education (higher vocational education, pre-university education, or university). More than half of the sample participants (55%; *n* = 92) was single, 16% (*n* = 27) was in a relationship, 24% (*n* = 40) was married, 5% (*n* = 8) was divorced, and one participant was widowed. See Table 1 for the demographic and clinical characteristics of the sample. Diagnoses of Axis-I disorders included major depressive disorder, dysthymia, generalized anxiety disorder, and others (see Table 1).

### 3.2. Preliminary item analysis and reliability

In the results from the correlation matrix (10 items), we identified no item pairs with correlations >0.85. However, when evaluating the item-rest correlations, it appeared that the total reliability improved with the removal of item 8 (“In the past week, could you stop these mental images of suicide if you wanted to?”) and item 10 (“In the past week, did you feel like you had to have such mental images of suicide, like a compulsion you couldn't escape?”). See Table 3 for SINAS item-test and item-rest correlations. While the 10-item scale had a Cronbach's α of 0.91, the eight-item scale had good internal reliability with a Cronbach α of 0.93. Given the importance of both items evaluating “controllability” and “compulsiveness” in combination with only a small improvement, we decided to keep the items. Therefore, going forward, we conducted the exploratory factor analysis with all ten items.

**Table 3:** Item-total and item-rest SINAS correlations table in a clinical population (*N* = 164).

	**Item-test correlation**	**Item-rest correlation**	**Average interitem correlation**	**Cronbach's alpha**
Item 1	0.87	0.82	0.48	0.89
Item 2	0.67	0.59	0.52	0.91
Item 3	0.70	0.62	0.51	0.90
Item 4	0.86	0.82	0.48	0.89
Item 5	0.80	0.74	0.49	0.90
Item 6	0.89	0.86	0.47	0.89
Item 7	0.86	0.81	0.48	0.89
Item 8	0.43	0.32	0.57	0.92
Item 9	0.77	0.70	0.50	0.90
Item 10	0.59	0.49	0.54	0.91
Test scale			0.50	0.91

### 3.3. Exploratory factor analysis

An exploratory factor analysis was performed using 164 participants, proving a ratio of 16.4 participants per item (26). The KMO score measure of sampling adequacy was 0.90, indicating that the data matrix was valid for the factor analysis (32). Furthermore, Bartlett's test of sphericity was significant [χ^2^(45) = 1124.78, *p* < 0.001], indicating that the variables were not too intercorrelated.

**Figure 1:**
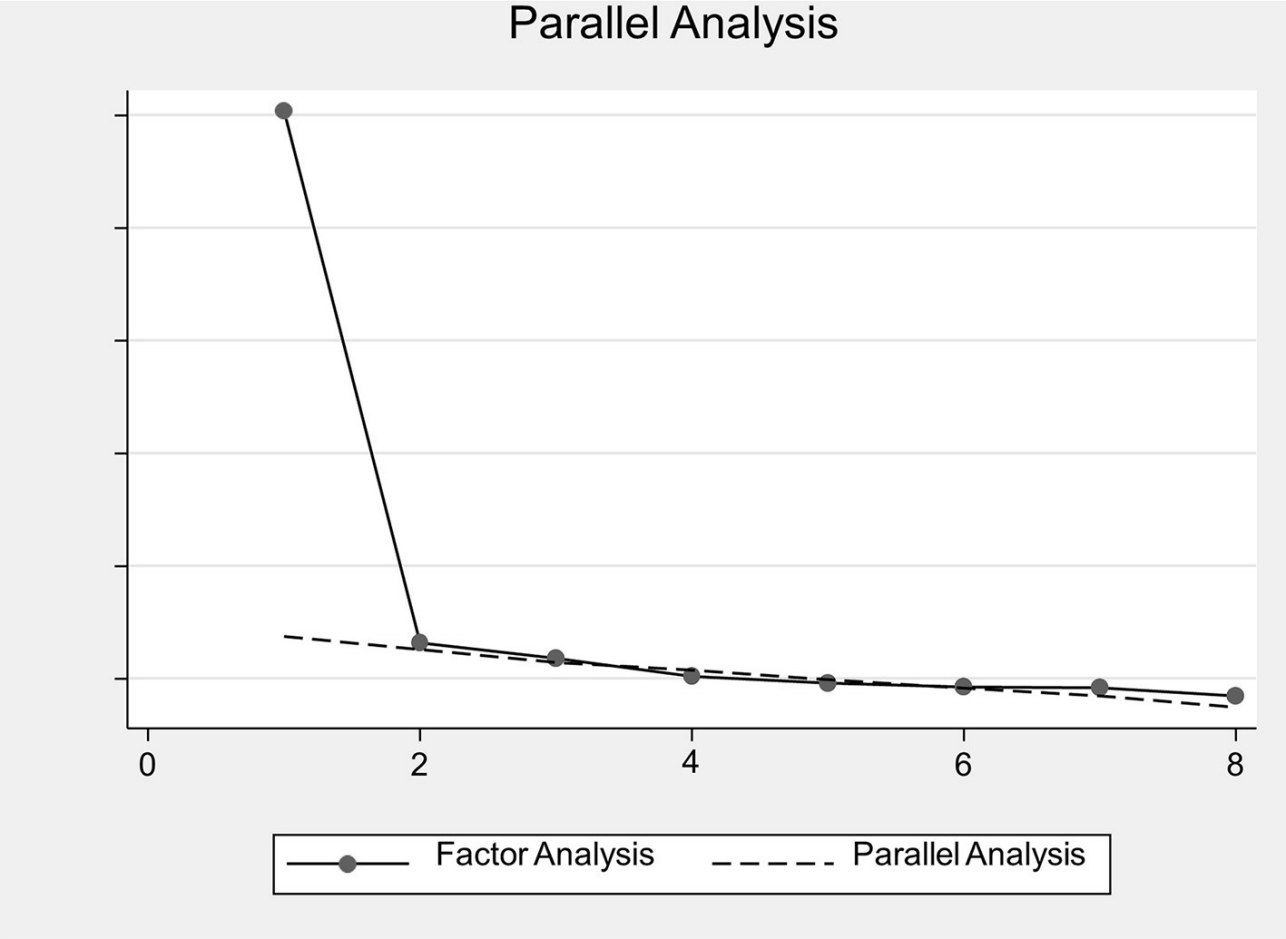
SINAS instrument scree plot from EFA and parallel analysis. EFA, exploratory factor analysis; SINAS, Suicidal Intrusions Attributes Scale.

One eigenvalue of > 1 was identified, and the parallel analysis confirmed this. The screen plot also pointed to the presence of one factor (see Figure 1). The rotated factor loadings (pattern matrix) for the one-factor EFA of the SINAS in the clinical sample can be found in Table 2.

### 3.4. Convergent and divergent validity SINAS

To assess the convergent validity, the Pearson correlation coefficients between the SINAS, SCI, and SIDAS were calculated. First, the SINAS and SCI showed a moderate correlation [r(155) = 0.43, *p* < 0.001]. Next, the SINAS and the SIDAS also showed a moderate correlation [r(162) = 0.59, *p* < 0.001].

**Figure 2:**
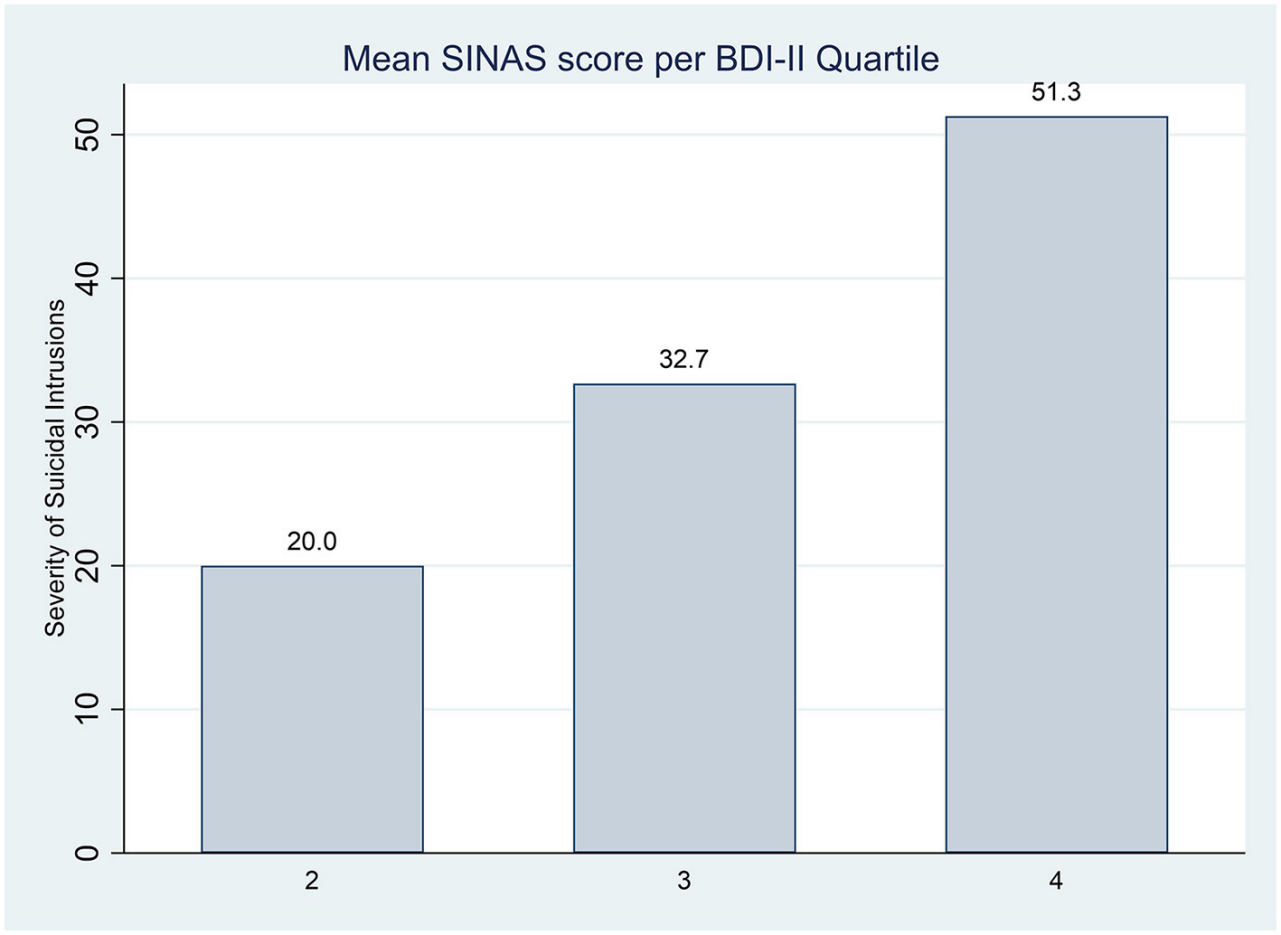
Mean SINAS scores divided into BDI-II Quartile range groups. BDI-II, Beck Depression Inventory—Second Edition; SINAS, Suicidal Intrusions Attributes Scale. BDI-II Quartile ranges: 2, mild to moderate range (10–18); 3, moderate to severe (19–29); 4, severe depression (30–63).

To assess the divergent validity, the Pearson correlation between the SINAS and the PIT and BDI-II was calculated. There was no significant correlation between the SINAS and the PIT [r(158) = 0.11, *p* = 0.16], indicating that individual differences in prospective mental imagery of non-suicidal aversive events are not associated with differences in suicidal intrusions. There was a distinct relationship between symptoms of depression and the SINAS [r(160) = 0.48, *p* < 0.001]. The more depressed the individual, the higher the SINAS scores and vice versa. See Figure 2 for the mean SINAS scores divided into BDI-II Quartile range groups, where the BDI-II Quartiles ranged from 2 = mild to moderate range (10–18), 3 = moderate to severe (19–29), to 4 = severe depression (30–63).

## 4. Discussion

The aim of this study was to develop and validate a self-reported questionnaire for suicidal intrusions that could be used in both research and clinical settings. In the current sample of outpatients with depression and suicidal ideation, the Suicidal Intrusions Attributes Scale (SINAS) showed good internal consistency and acceptable convergent and divergent validity. Moreover, the exploratory factor analysis (EFA) resulted in a single-factor structure.

Interestingly, we did not find high correlations between our instrument and the mental imagery scale (Prospective Imagery task). This indicates that the SINAS is a tool that distinguishes suicidal imagery and risk from everyday mental imagery experiences such as the tendency to use imagery or imagine the future in general in other areas. The suicidal ideation scale (SIDAS) showed a stronger correlation with the SINAS than with the other suicidal imagery instrument (Suicidal Cognition Interview). One possible explanation is the similarity in formulations between both instruments. The SINAS was developed and based on the methods of the SIDAS and its five attributes and was co-created by one of its authors. Therefore, the similarity in items, structure, and answer method (10-point Likert scale) could potentially affect the correlation between the instruments while still measuring different constructs. Another possible explanation is that the weaker correlation between the SINAS and the other suicidal imagery instrument could be explained by the chosen instrument. For the SCI, a semi-structured interview that has been used in multiple studies (1, 2, 18, 19), we limited the use of this instrument to only four relevant items assessing overall experiences with suicidal mental imagery (excluding items related to content or characteristics of the most important image). Therefore, it might not encompass or include all relevant items of the interview that would be needed to sketch a clear picture of the experienced suicidal mental imagery.

### 4.1. Strengths and limitations

At present, self-report measures of mental imagery-based suicidal thinking are lacking, despite the strong theoretical rationale that, compared to thinking in words, mental imagery has a more powerful impact on emotion and behavior, and thus, suicidal future-oriented images could increase suicide risk. An important strength of this study is testing in a suicidal clinical sample with a large sample size, which provided the recommended minimum sample size of 5–10 participants per item (19).

A limitation that needs to be considered is that potentially the number of items, chosen due to the brevity of the instrument, was too low to find factors for all the characteristics/elements involved in assessing the severity of suicidal intrusions. Notably, removing item 10 (“did you feel like you had to have such mental images of suicide, like a compulsion you couldn't escape?”) slightly increased the internal reliability of the overall instrument. However, this item was the only included item that provides important information on the compulsiveness of suicidal imagery.

### 4.2. Clinical implications and recommendations for future (psychometric) research

Observations of intrusive mental images related to suicide (flash-forwards) have been associated with past suicide attempts and can predict the worsening of suicidal ideation over time (11). Therefore, the SINAS can be used as a quick and easy-to-administer assessment tool for research and clinical settings. It may contribute to a richer overall assessment of suicide risk and its management by including mental imagery-based suicidal intrusions. During psychotherapy, it may assess suicidal images and triggers for these images as treatment targets, or for assessing post-treatment reductions in distressing suicidal images. Importantly, the tool is not only meant to diagnose a patient on the increased intrusiveness of suicidal imagery. Rather, it is meant to start a conversation with the patient regarding this topic and to review all aspects of the suicidal process.

Furthermore, our sample comprised a depressed clinical population, so we explored the possible relationship between depressive symptoms and the severity of suicidal intrusions. The results indicated a clear association, meaning the more depressed the individual, the higher the SINAS scores and vice versa. This could have important clinical implications since, as depressed individuals put a lot of energy into negative future-oriented suicidal intrusions, there might be little room for hopeful alternatives. If images about future are mainly occupied with one's own suicide, feelings of seeing a better, happier future and acting on these positive images to promote wellbeing may decrease (34).

Screening and assessing the presence and severity of suicidal intrusions is only in its infancy. To date, mostly non-validated time-consuming instruments have been used to evaluate suicidal intrusions (1, 20). This study has shown a substantial contribution of the SINAS in assessing suicidal intrusions in both clinical and research settings, but several key directions for future research remain. First, future studies may look at testing the replication of these findings in a larger sample size. Most importantly, the current one-factor structure provides the theoretical structure for an a priori hypothesis in a confirmatory factor analysis and additional testing (26, 35). Second, the brief questionnaire should be tested in other population samples to examine whether the instrument translates to samples with more variations in their suicidal ideation. Despite our sample being clinically diverse, the participants were predominantly receiving treatment for major depressive disorder and experienced high rates of suicidal ideation. Third, the SINAS does not distinguish between suicidal intrusions and similar phenomena observed in OCD. Future research may further elaborate on these differences. Finally, future longitudinal studies could evaluate to what extent the SINAS can be used to identify cutoff scores with imminent suicide risk or behavior. This could aid a therapist to use the screening tool to observe whether suicidal intrusions should be among an immediate or key focus of treatment, such as for those suffering from high levels of suicidal flash-forwards.

## 5. Conclusion

Research has shown that recalling mental images can have more powerful effects on emotional and physiological responses than verbal thinking (6, 15). The present study presents a novel yet first key step toward identifying and implementing validated screening and assessment tools for routine clinical suicide risk assessments. The SINAS is easy to administer, and no trained clinicians are required. Increasing routine assessments of suicidal intrusions in clinical assessment and the use of quick and effective screening tools may facilitate the understanding of suicidal mental imagery in patients, as well as further research. This, in turn, could be a stepping stone to developing alternative treatments for preventing suicide, as we are currently investigating the effectiveness of an add-on intervention (Eye Movement Dual Task; EMDT) in reducing suicidal intrusions in an RCT (27).

## Data availability statement

The raw data supporting the conclusions of this article will be made available by the authors, without undue reservation.

## Ethics statement

The studies involving human participants were reviewed and approved by the Medical Ethical Review Committee of the Amsterdam UMC (Protocol Number 2017.237). The patients/participants provided their written informed consent to participate in this study.

## Author contributions

JB did the statistical analysis. All authors wrote the first draft or provided input to the first draft of the report (JB, SG, MH, AK, and MS). All authors contributed to the article and approved the submitted version.
