# Supplementary material for: The Suicidal Intrusions Attributes Scale (SINAS): a new tool measuring suicidal intrusions
Source: Front Psychiatry. 2023 Jul 5;14:1158340. doi: 10.3389/fpsyt.2023.1158340 (PMC10354241; doi:10.3389/fpsyt.2023.1158340)
Supplement: Supplementary file 1 [file Table_1.docx]

Supplementary Material

The Suicidal Intrusions Attributes Scale (SINAS): A new tool measuring suicidal intrusions

# Jaël S. van Bentum^1,2*^, Marit Sijbrandij^1,3^, Ad Kerkhof^1^, Emily A. Holmes^4^, Stephan de Geus^1^, and Marcus J.H. Huibers^2,5^

**Table S1. Correlation Matrix 10-item Suicidal Intrusions Attributes Scale (SINAS)**

|  | 1 | 2 | 3 | 4 | 5 | 6 | 7 | 8 | 9 | 10 | 11 |
| --- | --- | --- | --- | --- | --- | --- | --- | --- | --- | --- | --- |
| 1. Item 1 | – |  |  |  |  |  |  |  |  |  |  |
| 2. Item 2 | .55^**^ | – |  |  |  |  |  |  |  |  |  |
| 3. Item 3 | .55^**^ | .38^**^ | – |  |  |  |  |  |  |  |  |
| 4. Item 4 | .79^**^ | .51^**^ | .54^**^ | – |  |  |  |  |  |  |  |
| 5. Item 5 | .67^**^ | .53^**^ | .60^**^ | .70^**^ | – |  |  |  |  |  |  |
| 6. Item 6 | .82^**^ | .55^**^ | .44^**^ | .81^**^ | .67^**^ | – |  |  |  |  |  |
| 7. Item 7 | .75^**^ | .51^**^ | .49^**^ | .70^**^ | .61^**^ | .87^**^ | – |  |  |  |  |
| 8. Item 8 | .25^*^ | .27^**^ | .23^*^ | .32^**^ | .33^**^ | .28^**^ | .29^**^ | – |  |  |  |
| 9. Item 9 | .59^**^ | .41^**^ | .52^**^ | .59^**^ | .49^**^ | .69^**^ | .78^**^ | .22^*^ | – |  |  |
| 10. Item 10 | .47^**^ | .30^**^ | .41^**^ | .45^**^ | .38^**^ | .48^**^ | .37^**^ | .28 | .42^**^ | – |  |
| 11. Total | **.86^**^** | **.67^**^** | **.70^**^** | **.86^**^** | **.80^**^** | **.90^**^** | **.86^**^** | **.44^**^** | **.77^**^** | **.59^**^** | **–** |

*Note. N* = 164. ^*^*p* < .01, ^**^ *p* < .001
