# Supplementary material for: The Suicidal Intrusions Attributes Scale (SINAS): a new tool measuring suicidal intrusions
Source: Front Psychiatry. 2023 Jul 5;14:1158340. doi: 10.3389/fpsyt.2023.1158340 (PMC10354241; doi:10.3389/fpsyt.2023.1158340)
Supplement: Supplementary file 2 [file Data_Sheet_1.docx]

Supplementary Material

The Suicidal Intrusions Attributes Scale (SINAS): A new tool measuring suicidal intrusions

# Jaël S. van Bentum^1,2*^, Marit Sijbrandij^1,3^, Ad Kerkhof^1^, Emily A. Holmes^4^, Stephan de Geus^1^, and Marcus J.H. Huibers^2,5^

# Supplementary Data

**S1. Dutch items with answer marking Suicidal Intrusions Attributes Scale (SINAS)**

Explanatory text before questions: “De volgende vragen gaan over het hebben van mentale beelden. Soms denken we in de vorm van woorden en zinnen (‘verbale gedachten’), en soms denken we meer in mentale beelden. Dit zijn gedachten die door je hoofd kunnen gaan in de vorm van beelden of plaatjes. Wilt u de onderstaande vragen beantwoorden over de afgelopen week? Wilt u de vragen beantwoorden met een rapportcijfer? (0 = helemaal niet of nooit, 10 = voortdurend, in zeer sterke mate).”

| Vraag | 0 | 10: |
| --- | --- | --- |
| 1. In de afgelopen week, hoe vaak had u beelden over uw eigen zelfmoord? | Helemaal niet | Voortdurend |
| 2. In de afgelopen week, hoeveel controle ervoer u over deze suicidale beelden? | Volledige controle | Geen controle |
| 3. In de afgelopen week, hoe dicht was u bij een zelfmoordpoging? | Helemaal niet dichtbij | Ik heb een zelfmoordpoging gedaan |
| 4. In de afgelopen week, in welke mate werd u gekweld door beelden over zelfmoord? | Helemaal niet gekweld | In zeer sterke mate gekweld |
| 5. In de afgelopen week, in welke mate hebben beelden aan zelfmoord u gehinderd in het uitvoeren van uw dagelijkse bezigheden zoals werk, huishouden, en sociale activiteiten? | Helemaal niet gehinderd | In zeer sterk mate gehinderd |
| 6. In de afgelopen week, hoe indringend waren de beelden over zelfmoord die u zag? | Helemaal niet indringend | In zeer sterk mate indringend |
| 7. In de afgelopen week, hoe levendig waren de beelden aan zelfmoord die u zag? | Helemaal niet levendig | In zeer sterk mate levendig |
| 8. In de afgelopen week, kon u deze beelden stoppen als u dat wilde? | Helemaal niet | Volledig |
| 9. In de afgelopen week, had u beelden die zo helder waren dat het leek alsof die beelden werkelijkheid waren? | Helemaal niet | In zeer sterke mate |
| 10. In de afgelopen week had u het idee dat u dergelijke beelden moest hebben, als een dwang waaraan u niet kon ontkomen? | Helemaal niet | In zeer sterke mate |
